# Myopia Development in Tree Shrew Is Associated with Chronic Inflammatory Reactions

**DOI:** 10.3390/cimb44090296

**Published:** 2022-09-17

**Authors:** Hsiangyu Ku, Jamie Jiin-Yi Chen, Min Hu, Peng-Tai Tien, Hui-Ju Lin, Gezhi Xu, Lei Wan, Dekang Gan

**Affiliations:** 1 Department of Ophthalmology and Visual Science, Eye and ENT Hospital, Shanghai Medical College, Fudan University, Shanghai 200031, China; 2 Eye Center, China Medical University Hospital, Taichung 404333, Taiwan; 3 Department of Pediatric Ophthalmology, Hospital of Yunnan University, Kunming 650091, China; 4 Graduate Institute of Clinical Medical Science, College of Medicine, China Medical University, Taichung 404333, Taiwan; 5 School of Chinese Medicine, China Medical University, Taichung 404333, Taiwan; 6 Shanghai Key Laboratory of Visual Impairment and Restoration, Shanghai 200031, China; 7 Key Laboratory of Myopia of State Health Ministry, Shanghai 200031, China; 8 Department of Biotechnology, Asia University, Taichung 404333, Taiwan; 9 Department of Obstetrics and Gynecology, China Medical University Hospital, Taichung 404333, Taiwan

**Keywords:** myopia, inflammation, tree shrew, form deprivation

## Abstract

In this study, we aimed to investigate whether chronic retinal inflammation is involved in the pathogenesis of form-deprivation myopia (FDM) using tree shrews as an animal model. Twenty-one tree shrews were randomly divided into 7-day/14-day FDM (FDM7/FDM14) groups and their corresponding 7-day/14-day control groups. Refraction and axial length were measured. To determine the effects of form deprivation on inflammation, we used real-time polymerase chain reaction (PCR) and immunohistochemistry to assess the expression levels of several proinflammatory cytokines. At day 0, the eyes in the FDM and control groups were hyperopic. However, after 7 and 14 days of form deprivation, the refractive error of the eyes in the FDM7 and FDM14 groups shifted from +6.6 ± 0.3 diopters (D) to +4.0 ± 0.5 D and from +6.4 ± 0.3 D to +5.0 ± 0.3 D, respectively. The levels of tumor necrosis factor-α, interleukin (IL)-6, IL-8, monocyte chemoattractant protein-1, and nuclear factor κB were increased in the FDM eyes, compared with those in the control eyes. The increase in matrix metalloproteinase-2 expression was greater in the FDM eyes than in the contralateral and control eyes, whereas collagen type I expression was downregulated. In conclusion, chronic inflammation may play a crucial pathogenic role in form-deprivation myopia in tree shrews.

## 1. Introduction

Myopia is a frequent cause of irreversible visual disorders, particularly in younger populations in East and Southeast Asia. With a projected incidence of 50% worldwide by 2050, myopia may become the primary cause of irreversible blindness [1]. Numerous studies have demonstrated that environmental factors, such as differences in outdoor and indoor activities [2] and longer near-work times [3], influence the development of myopia. Myopia is generally managed by spectacle lenses, while contact lenses or refractive laser surgery are also common. These measures aim to correct the mismatch between the ocular axial length (AL) and the ocular refractive error (RE), and thereby restore normal vision. Despite strict adherence to established treatment plans and appropriate public advocacy, the incidence of myopia is still climbing. Patients whose myopia progresses rapidly to high myopia may experience other complications, such as cataracts, glaucoma, retinal detachment, and myopic maculopathy, that may result in blindness [4]. Thus, the pathogenesis underlying myopia needs to be elucidated to allow the development of interventions that may help to prevent the complications of myopia and blindness.

Since the first report describing an animal model of myopia [5], numerous animal studies have demonstrated that excessive axial elongation in myopic eyes is associated with scleral remodeling, scleral tissue loss involving decreased connective tissue synthesis, and the increased degradation of collagen type I (COL-I), which ultimately lead to adverse changes in the composition of the sclera and its ductility [6,7]. Moreover, many recent studies have addressed the molecular and genetic mechanisms contributing to the progression of myopia and have shown that the retina–sclera signaling cascade leads to the growth and remodeling of the eye [8]. The signal that activates ocular tissue remodeling is derived from the retina and involves a series of reactions in photoreceptors and retinal pigment epithelium (RPE). The RPE possesses many signal transduction receptors and molecules that regulate eye growth, particularly transforming growth factor-β (TGF-β), insulin-like growth factor-1, and fibroblast growth factor. Depending on the direction of secretion, these growth factors may affect the growth of the inner retina, choroid, or sclera [9].

Several reports have proposed that inflammation plays a role in susceptibility to myopia [10,11,12,13]. Indeed, a study of patients with autoimmune diseases, such as systemic lupus erythematosus, uveitis, or type 1 diabetes mellitus, revealed that their risks of developing myopia are greater than those of people without these diseases. Moreover, the levels of inflammatory markers such as c-Fos, nuclear factor κB (NF-κB), interleukin (IL)-6, and tumor necrosis factor (TNF)-α were upregulated in myopic eyes in an animal model (Lin et al. 2016; Wei et al. 2018). Thus, both clinical and experimental studies have demonstrated the pathogenic effects of inflammation in the progression of myopia.

Many early studies of myopia used chickens as experimental subjects, but their anatomical structures are very different from those of mammals. Although rodents have also been widely used, the rodent retina is dominated by rod cells, and the rodent visual system differs from that of humans. By contrast, the tree shrew (*Tupaia belangeri*) is a small mammal with well-developed binocular and color vision, and the eyes have an all-fibrous sclera that is closely related to that of the primates [14]. Emmetropization is often believed to regulate eye growth but not always toward emmetropia. Similar to human eyes, the eyes of tree shrews grow toward low hyperopia [15]. The anatomic and physiological similarities between tree shrews and other species also indicate that their direct emmetropization signaling cascades are similar. Thus, in our study, we investigated whether the tree shrew model of FDM could adequately characterize axial elongation and its association with chronic inflammation.

## 2. Materials and Methods

### 2.1. Animal Model of Myopia

Tree shrews (*Tupaia belangeri chinensis*) were obtained from the Kunming Institute for Research on Zoology, the Chinese Academy of Sciences, China. Twenty-one tree shrews were randomly allocated into 7-day/14-day FDM (FDM7/FDM14) groups and their corresponding 7-day/14-day control groups. Twenty-four days after the first day of visual experience (24 ± DVE) [16], the animals were anesthetized with 3% sodium pentobarbital (0.1 mL/150 g) administered intraperitoneally. We sutured the lids of one eye, as previously described [17], and the contralateral eye was used as self-control. Table 1 shows the length of treatment for each group. The animals were housed in temperature-controlled rooms in the animal facilities at the Kunming Medical School, at room temperature (23 ± 2 °C) with a relative humidity of 70–80% and a 12 h dark/light cycle. One animal was placed in each cage. All the tree shrews had free access to standard food and water, and fresh milk was supplied daily. Milk, eggs, silkworm chrysalises, and fruits were given as supplements to the standard diet. The animals were treated and cared for in accordance with the ARRIVE guidelines [18]. All studies were approved by the Committee for Animal Welfare of the Eye and ENT Hospital of Fudan University.

### 2.2. Physiological Measurements and Tissue Preparation

After assigning an identification number to each animal, the pupils were dilated (0.5% proxymetacaine hydrochloride; Alcon, Bornem, Belgium), and the cornea was anesthetized (4% oxybuprocaine hydrochloride; Santen, Osaka, Japan). The cycloplegic refractive error of both eyes was examined using a streak retinoscope (Keeler, Windsor, UK) in a dark room. Then, A-scan ultrasonography (11 MHz; Optikon HiScan A/B) was performed to measure the lens thickness (LT), axial length (AL), and vitreous chamber depth (VCD). At the end of the myopia induction period, the animals were euthanized using an overdose of sodium pentobarbital, and the eyes were enucleated. Under a surgical microscope (Carl Zeiss, Aalen, Germany), we used a razor blade to collect the tissues on an ice-cold plate by making a cut on the eye perpendicular to the anterior or posterior axis at a location approximately 1 mm posterior to the ora serrata. We then separated the iris and ciliary body of the anterior segment. The retinas were dissected in ice-cold phosphate-buffered saline (PBS) and immersed in an Allprotect Tissue Reagent (Qiagen, Valencia, CA, USA). All the tissue samples were stored at −80 °C prior to RNA extraction.

### 2.3. Immunohistochemistry

Eyes were collected from each animal, embedded in paraffin, and cut at a thickness of 8 μm. The resulting sections were collected on glass slides. The sections were deparaffinized, immersed in hydrogen peroxide (30 min), blocked in PBS containing 5% normal goat serum (1 h at room temperature), and incubated (overnight at 4 °C) with specific primary antibodies against COL-I (GeneTex Cat# GTX20292 RRID:AB_384293), IL-6 (Abcam Cat# ab6672 RRID:AB_2127460), IL-8 (MyBioSource Cat# MBS551025), matrix metalloproteinase-2 (MMP2) (Abcam Cat# ab37150 RRID:AB_881512), monocyte chemoattractant protein-1 (MCP-1), NF-κB (Abcam Cat# ab16502), TGF-β (Abcam Cat# ab66043 RRID:AB_1143428), (Abcam Cat# ab9669 RRID:AB_2071551), and TNF-α (Bioworld Cat# BS1857). Next, the tissues were incubated with an appropriate biotin-conjugated secondary antibody (1 h at room temperature). To develop the slides, streptavidin-conjugated horseradish peroxidase with diaminobenzidine (Sigma-Aldrich, WI, USA) was used as the substrate and counterstained with hematoxylin. ImageJ 1.46 r software was used for band densitometry and to determine the relative expression levels of each protein.

### 2.4. Quantitative Polymerase Chain Reaction (PCR)

The total RNA was extracted from the retinas using RNeasy Mini Kits (Qiagen, Valencia, CA, USA), and RNA (5 μg) was reverse-transcribed to cDNA using the Superscript First Strand Synthesis system (Invitrogen, Carlsbad, CA, USA). The primers and probes used in PCR were retrieved from the Universal Probes Library (Roche, West Sussex, UK). The transcript levels were normalized by the transcript level of glyceraldehyde 3-phosphate dehydrogenase.

### 2.5. Statistical Analysis

Data were statistically analyzed using Prism version 8 (GraphPad, San Diego, CA, USA) and were calculated as the mean ± standard error of the mean or as immunohistological scores. Student’s *t*-tests were used to compare the differences between the control and FDM groups, and the differences between subgroups were evaluated by one-way analysis of variance (ANOVA) for a completely randomized experimental design. The level of statistical significance was set at *p* < 0.05.

## 3. Results

The ocular characteristics of the FDM7, FDM14, and corresponding control (CON7 and CON14) groups are summarized in Table 1. There were no differences between the FDM and control groups in the VCD, AL, and RE at the baseline. At day 0, the tree shrews in the FDM and control groups were all hyperopic. However, after 7 and 14 days of form deprivation, the refractive error in the FDM7 and FDM14 groups’ treatment eye decreased from +6.6 ± 0.3 diopters (D) to +4.0 ± 0.5 D and from +6.4 ± 0.3 D to +5.0 ± 0.3 D, respectively.

**Figure 1:**
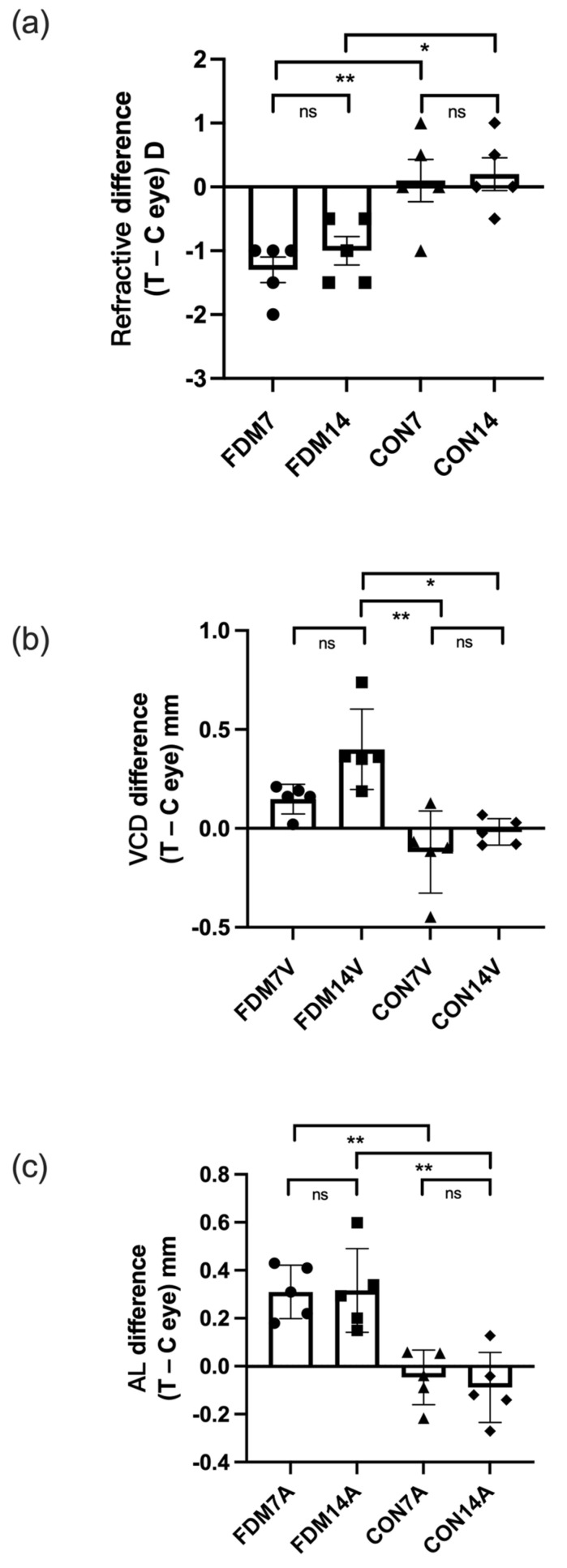
Differences in ocular refraction (**a**), vitreous chamber depth (**b**), and AL (**c**) between the treated eye and contralateral control eye (left and right eyes in the CON7 and CON14 groups) in the FDM and control groups. The three parameters indicated significant myopia in the FDM eyes compared with the CON7 and CON14 eyes. Values are shown as the mean ± standard error. * *p* < 0.05, ** *p* < 0.01. C, contralateral control eye; ns, not significant; T, treated eye; VCD, vitreous chamber depth.

To further examine the differences between the FDM eye and the contralateral control eye, we calculated the ocular parameters in the FDM groups as the difference between the treated and self-control eyes, and the difference between the right and left eyes in the control groups. Figure 1 shows the refraction, VCD, and AL in the four groups. There were significant differences in the refractive error between the CON7 group and the FDM7 and FDM14 eyes at the end of the treatment (ANOVA, *p* < 0.05). The VCD in the FDM14 eyes was significantly different from those in the CON7 and CON14 groups (ANOVA, *p* < 0.05). Moreover, the ALs in the FDM7 and FDM14 groups were significantly different from those in the CON7 and CON14 groups, respectively.

**Figure 2:**
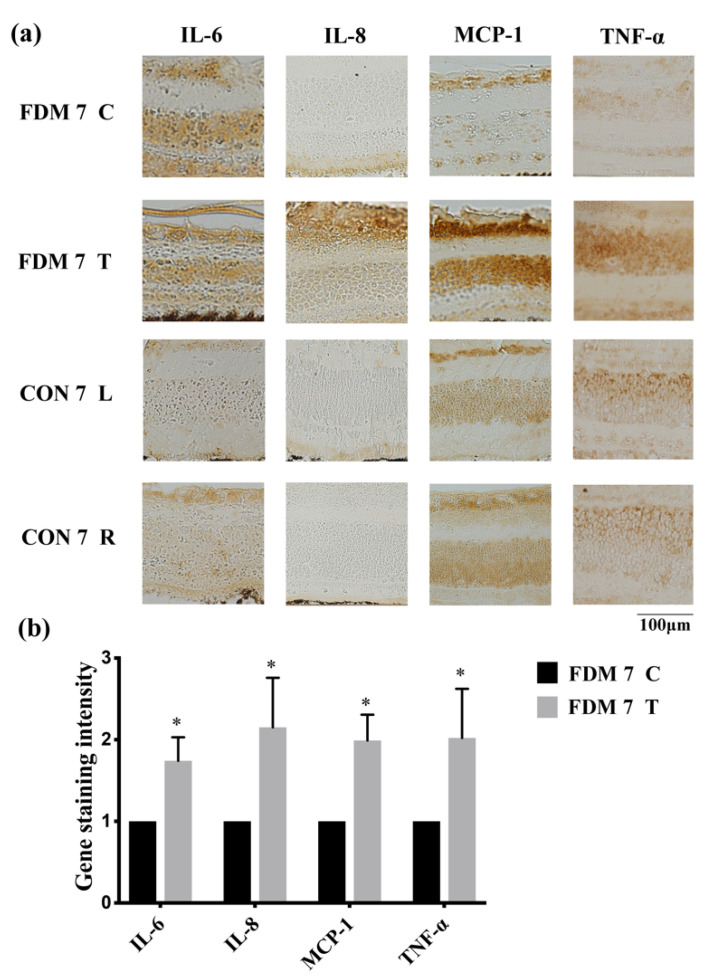
Expression levels of proinflammatory transcription factors and cytokines in the FDM and control eyes: (**a**) immunohistochemical analysis of IL-6, IL-8, MCP-1, and TNF-α expression at day 7 in the FDM and control groups; (**b**) gene staining intensity of IL-6, IL-8, MCP-1, and TNF-α determined using ImageJ software. * *p* < 0.05 (paired *t*-tests). ns, not significant; T, treated eye; C, contralateral control eye; L, left eye; R, right eye.

**Figure 3:**
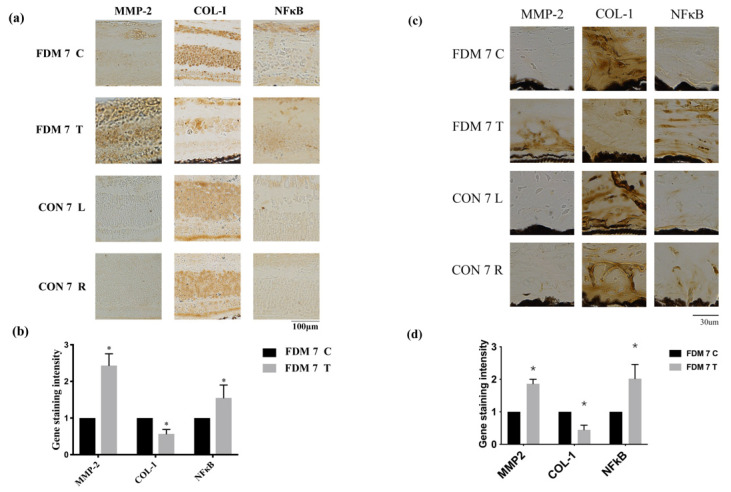
Expression levels of tissue remodeling proteins and inflammatory molecules increased in the FDM and control eyes: (**a**) immunohistochemical analysis of MMP2, NF-κB, and COL-I expression at day 7 in the FDM and control groups in the retina; (**b**) gene staining intensity of MMP2, NF-κB, and COL-I in the retina determined using Image J software.; (**c**) immunohistochemical analysis of MMP2, NF-κB, and COL-I expression at day 7 in the FDM and control groups in the sclera; (**d**) gene staining intensity of MMP2, NF-κB, and COL-I in sclera determined using Image J software. * *p* < 0.05 (paired *t*-tests).

To confirm whether FDM was associated with inflammation, we determined several proinflammatory cytokines using immunohistochemistry and real-time quantitative PCR. Immunohistochemical analysis revealed that TNF-α, IL-6, IL-8, and MCP-1 were upregulated in the FDM7 eyes (Figure 2). The retinal mRNA expression levels of TNF-α and IL-6 were significantly greater in the FDM7 eyes than in the contralateral eyes and the CON7 eyes (Figure 3a). Similarly, the retinal mRNA expression levels of TNF-α, IL-6, IL-8, and MCP-1 were significantly greater in the FDM14 eyes than in the contralateral eyes (Figure 3c).

**Figure 4:**
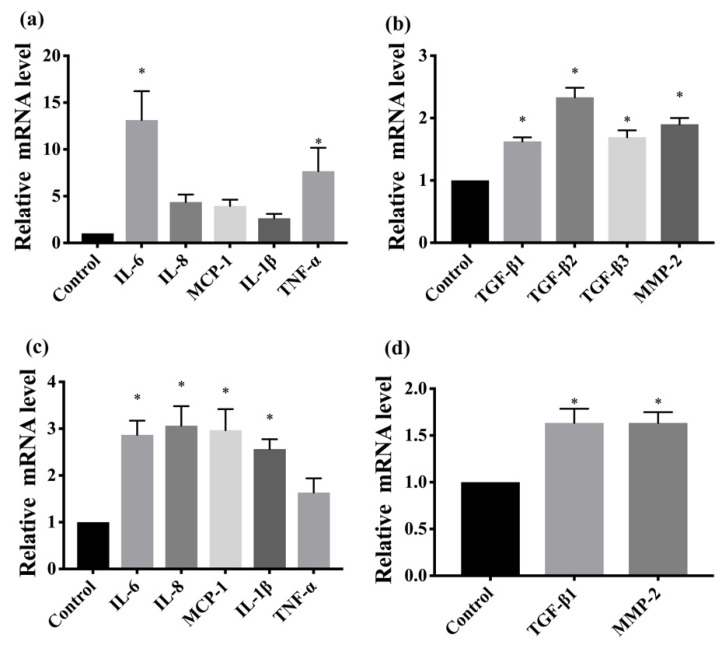
(**a**) Comparison of retinal mRNA expression levels of TNF-α, IL-6, IL-8, MCP-1, and IL-1β between the FDM7 and CON7 groups.; (**b**) comparison of retinal mRNA expression levels of TGF-β1, TGF-β2, TGF-β3, and MMP2 between the FDM7 and CON7 groups.; (**c**) comparison of retinal mRNA expression levels of TNF-α, IL-6, IL-8, MCP-1, and IL-1β between the FDM14 and CON14 groups.; (**d**) retinal mRNA expression levels of TGF-β1 and MMP2 between the FDM14 and CON14 groups. * *p* < 0.01 (ANOVA).

To confirm which signaling pathway is involved in the progression of myopia, we performed immunohistochemistry to determine the levels of MMP2, COL-1, and NF-κB. MMP2 and NF-κB were upregulated in the FDM7 eyes relative to the contralateral eyes and the CON7 eyes, whereas COL-I expression was downregulated in the FDM7 eyes (Figure 4). The mRNA expression levels of TGF-β1, TGF-β2, TGF-β3, and MMP2 were significantly greater in the FDM7 eyes than in the contralateral eyes (Figure 3b). The mRNA expression levels of TGF-β1 and MMP2 were significantly greater in the FDM14 eyes than in the contralateral eyes (Figure 4d).

## 4. Discussion

Few studies have investigated whether inflammation is associated with the progression of myopia. During inflammation, a variety of cytokines, prostaglandins, blood cells, and growth and cytotoxic factors are attracted to the site of infection or injury, and blood is also redirected to the affected site [19]. This vital process may initiate tissue repair, because the tissue factors are involved in converting fibroblasts to myofibroblasts and induce local biochemical reactions to facilitate tissue remodeling [20,21]. Similar structural modifications occur in myopic eyes, suggesting a pathogenic role of inflammation in the progression of myopia. Supporting this concept, we recently described a correlation between myopia and allergic conjunctivitis (AC), a relatively chronic inflammatory disease [11,13].

Tree shrews are mammals with excellent vision and an all-fibrous sclera similar to that of humans. The similarities between tree shrews and other species also suggest similarities in the direct emmetropization signaling cascade. However, no studies have elucidated the effects of chronic inflammation on FDM in tree shrews. Therefore, we examined the relationship between chronic inflammation and FDM, as a model of myopia, in tree shrews.

The refractive error is detected by retinal amacrine cells [22], which generate the signals that travel via a direct signaling pathway to the RPE and traverse the choroid [23] to modify sclera extracellular matrix (ECM) remodeling [24] to facilitate myopic eye growth [25,26,27]. A genetic linkage study identified several candidate genes that encode various components of the ECM growth and remodeling pathways [28]. Scleral thinning in highly myopic eyes is caused by a reduction in COL-I, the major structural protein expressed in the sclera [29]. The degradation of COL-I in myopic eyes was attributed to the increased synthesis of MMPs, including active MMP2 [7]. 

Consistent with earlier studies [29,30], we found that the TGF-β and MMP2 mRNA levels were greater in the FDM eyes of tree shrews than those in the self-control eyes and corresponding control groups, whereas COL-I expression was downregulated. MMPs perform several functions during tissue repair processes, particularly fibrosis. They immediately cause the disruption of the basement membrane, allowing inflammatory cells to reach the site of injury [31]. Furthermore, myofibroblasts control collagen and ECM protein turnover and remodeling through a pathway involving MMPs [32]. 

Our study also demonstrated an increase in the inflammatory response in the FDM7 and FDM14 eyes. The expression levels of TGF-β, TNF-α, IL-6, IL-8, and MCP-1 were increased in the myopic eyes in both FDM groups. These results are consistent with the outcomes that used a different FDM method and other animal species (guinea pigs and hamsters) [11]. MCP-1, TGF-β, and IL-8 activate NF-κB, which modulates inflammatory responses [33,34]. Similarly, MCP-1 expression was increased in the aqueous humor of patients with high myopic cataracts [35]. TNF-α and IL-8 are also involved in other ocular inflammatory disorders, such as endotoxin-induced uveitis [36,37]. In the NF-κB pathway, the signal from proinflammatory mediators may activate the inhibitor of NF-κB (IκB) kinase α/β, which phosphorylates IκB. The subsequent degradation of IκB leads to NF-κB activation, which increases the production of TNF-α, IL-6, and other proinflammatory cytokines. NF-κB can also activate the expression of MMP2 and TGF-β while downregulating COL-I expression to promote myopia progression. TGF-β is also a major component of the ECM and is directly involved in the regulation of collagen, proteoglycan, and MMPs [38]. The early changes in TGF-β expression may reflect its role in the retinoscleral signal pathway involved in myopic eye growth [39]. Overall, our findings indicate that FDM is associated with an inflammatory state, which is likely to facilitate the progression of myopia.

Numerous studies have shown that extended continuous reading is a risk factor for myopia [40,41]. Asthenopia, which is caused by extended eye activities, may lead to dryness, burning sensations, strain, ophthalmalgia, and blurred vision [42]. These chronic inflammatory symptoms could then promote myopia. Our previous study revealed a correlation between myopia and allergic inflammatory diseases. Children with inflammatory diseases, such as AC, atopic dermatitis, allergic rhinitis, or asthma were more likely to develop myopia, and the risk was greatest in children with AC [13]. The complement system is thought to promote ECM remodeling in the sclera during the development of myopia [43]. A meta-analysis revealed the transcriptional activation of the complement system during myopia and confirmed that several cell signaling pathways and mitochondrial and structural processes are involved in refractive errors [44]. Although the altered expression of structural genes is anticipated, when we consider the morphological features of refractive errors, it seems that most of the implicated immune-related genes belonged to the complement–coagulation pathway, which induces immune and inflammatory responses. More recently, it was reported that scleral hypoxia may promote myofibroblast transdifferentiation as collagen production declines [45]. The hypoxia-inducible factor-1α-controlled transcriptional regulation was also identified in extensive research into glycolytic metabolism, hypoxia, and the inflammatory responses that occur in neutrophils and macrophages, and it was proposed as a potential target capable of modulating inflammation [46]. This was further corroborated by our finding that inflammation may play an important role in the development of myopia.

In this study, we established an animal model of FDM in tree shrews to investigate whether inflammation is linked to the development of myopia. Myopia is a complex, multigenic disease under the influence of several overlapping signaling pathways. However, the pathogenic role of inflammation in myopia remains unclear. Further studies with larger sample sizes, in animal models and humans, are needed to validate our findings and to provide a more conclusive overview of the mechanisms involved in the pathogenesis of myopia.

## Figures and Tables

**Table 1:** Ocular biometrics and refraction.

Group	Day	AL, mm		Refraction, D	
		**TE**	**CE**	**TE**	**CE**
**FDM7 (n = 5)**	0	6.66 ± 0.04	6.62 ± 0.04	6.8 ± 0.37	6.6 ± 0.4
	7	7.02 ± 0.03	6.741± 0.02	4.5 ± 0.2	5.8 ± 0.33
**CON7 (n = 5)**	0	6.78 ± 0.03	6.88 ± 0.02	5.9 ± 0.29	5.6 ± 0.48
	7	6.89 ± 0.03	6.94 ± 0.05	5.0 ± 0.01	4.9 ± 0.3
**FDM14 (n = 5)**	0	6.68 ± 0.06	6.79 ± 0.09	6.2 ± 0.25	6.3 ± 0.3
	14	7.02 ± 0.10	6.80 ± 0.10	4.9± 0.36	5.9 ± 0.4
**CON14 (n = 5)**	0	6.70 ± 0.07	6.78 ± 0.06	5.5 ± 0.38	5.5± 0.38
	14	6.76 ± 0.07	6.78 ± 0.09	5.4 ± 0.24	5.5± 0.31

Ocular biometric data were determined by A-scan ultrasound and refractive data by streak retinoscopy. Data are expressed as the mean ± standard error.TE, treated eye; CE, contralateral control eye; FDM7, 7-day form-deprivation myopia group; CON7, 7-day control group; FDM14, 14-day form-deprivation group; CON14, 14-day control group.

## Data Availability

Not applicable.
